# Biological Age, Aging Clocks, and the Interplay with Lymphoid Neoplasms: Mechanisms and Clinical Frontiers

**DOI:** 10.3390/lymphatics3030019

**Published:** 2025-07-11

**Authors:** Xiaocan Wu, Hanna Liu, Kejun Ying

**Affiliations:** 1 Harvard T.H. Chan School of Public Health, Harvard University, Boston, MA 02115, USA; 2 College of Pharmacy, Fujian University of Traditional Chinese Medicine, Fuzhou 350108, China; 3 School of Pharmacy, Massachusetts College of Pharmacy and Health Sciences, Boston, MA 02115, USA

**Keywords:** aging, biological age, omics, lymphoid neoplasms

## Abstract

Lymphoid neoplasms (LN), a diverse group of malignancies arising from lymphocytes, exhibit a striking increase in incidence with chronological age, suggesting a deep connection with the aging process. While chronological age remains a primary risk factor, the concept of biological age, reflecting an individual’s physiological state and susceptibility to age-related diseases, offers a more nuanced understanding of this relationship. Aging clocks, particularly epigenetic clocks based on DNA methylation, provide quantitative measures of biological age and have revealed associations between accelerated aging and increased cancer risk, including LN. Immunosenescence, the age-related decline in immune function characterized by thymic involution, altered lymphocyte populations, and chronic inflammation (inflammaging), appears to be a key mechanistic link between aging and LN development, potentially providing a more accurate predictor of cancer risk than mutation accumulation alone. Accelerated biological aging, measured by various clocks, correlates with LN risk and progression (e.g., in chronic lymphocytic leukemia), and may influence treatment tolerance and outcomes, particularly in older adults who are often burdened by frailty and comorbidities like sarcopenia. Integrating biological age assessments into clinical practice holds promise for refining diagnosis, prognosis, and personalizing treatment strategies (including guiding intensity and considering anti-aging interventions), and improving outcomes for patients with LN. This review synthesizes the current understanding of the intricate relationship between LN, immunosenescence, biological age, and aging clocks, highlighting clinical implications and key future research directions aimed at translating these insights into better patient care.

## Graphical Abstract

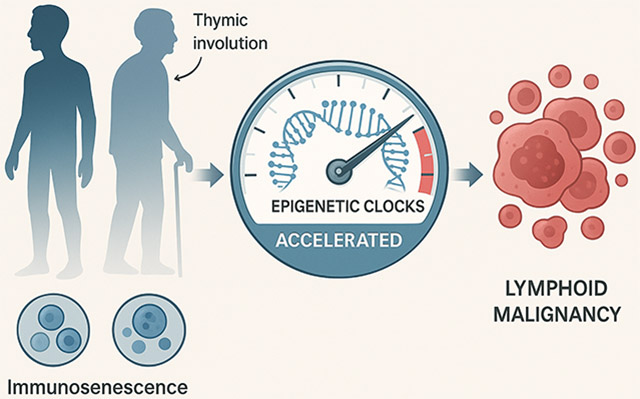

## 1. Introduction

Lymphoid neoplasms (LN) represent a heterogeneous collection of malignancies originating from B-lymphocytes, T-lymphocytes, or natural killer (NK) cells at various stages of differentiation [1,2]. Their incidence, particularly for subtypes like diffuse large B-cell lymphoma (DLBCL), has been increasing, and they predominantly affect older adults [1,3]. However, certain lymphoid neoplasms demonstrate unique age distributions. Hodgkin lymphoma (HL), for example, exhibits a bimodal age distribution with peaks in young adults (ages 15–40) and older adults (after age 55), suggesting that aging processes may influence lymphomagenesis differently across the age spectrum [4,5]. The strong association between chronological age and LN incidence points towards underlying biological aging processes playing a critical role in lymphomagenesis [6].

However, chronological age is an imperfect proxy for an individual’s physiological state. Biological age refers to the functional state of an individual’s body systems and their susceptibility to age-related diseases, which can differ substantially from chronological age [7,8]. Biological age reflects functional capacity and vulnerability to age-related decline and disease, often diverges from chronological age, and may provide a more accurate assessment of aging [7,9]. Over the past decade, significant advances have been made in developing various “aging clocks”: biomarkers or algorithms designed to quantify biological age [10,11]. Epigenetic clocks are computational models that use DNA methylation patterns at specific cytosine-guanine dinucleotide (CpG) sites to estimate biological age [12,13]. Among these, epigenetic clocks, based on age-related DNA methylation (DNAm) patterns, have emerged as particularly robust predictors [9,12,14-16].

Accelerated biological aging, where the biological age surpasses the chronological age, is associated with an increased risk for numerous age-related diseases and all-cause mortality [17,18]. Growing evidence now links accelerated biological aging, often measured by these clocks, specifically to the risk and progression of LN [19-21]. Recent work has shown that B cell lymphoma development is associated with age-associated clonal B cell populations driven by epigenetic changes and accelerated biological aging [22]. This review explores the complex interplay between LN development, the fundamental processes of aging (particularly immunosenescence), and the insights provided by both biological age and aging clocks. While our focus primarily centers on LN subtypes that predominantly affect older adults, we also consider the role of biological aging in lymphomas with unique age distributions, such as Hodgkin lymphoma. We discuss the underlying mechanisms, along with the clinical implications for diagnosis, prognosis, and treatment of LN, and outline future research directions needed to harness this knowledge for improved patient outcomes.

### 1.1. Lymphoid Neoplasms and the Impact of Chronological Aging

Lymphoid neoplasms exhibit distinct age-related incidence patterns. While precursor T- and B-cell malignancies peak in children and young adults, most LN derived from mature lymphocytes, particularly B-cells, show a steady increase in incidence with age, typically peaking between 75 and 99 years [6]. However, Hodgkin lymphoma represents a notable exception with its characteristic bimodal age distribution. This bimodal pattern may reflect “the varying proportions of cell populations across the age range, with an immune system rich in precursor cells in young people and a predominance of germinal centre and memory B-cells in older adults.” This pattern holds across various geographical regions, although the absolute rates differ [6]. This age-related distribution likely reflects changes in the composition and function of the immune system over the lifespan. Interestingly, some studies suggest a potential decline or plateau in incidence and mortality in the very oldest cohorts (e.g., >80–85 years), possibly linked to factors like decreased lymphopoiesis or impaired angiogenesis limiting tumor growth in extreme old age [23,24]. However, the overwhelming trend is a significantly higher burden of LN in the elderly population [25].

### 1.2. Immunosenescence: A Key Mechanistic Link

The age-related increase in LN is strongly linked to immunosenescence: the multifaceted deterioration of immune function with age [26,27]. Like an aging security system that becomes less effective at detecting threats, immunosenescence leaves the body vulnerable to both infections and malignant transformations. This process impacts both innate and adaptive immunity, leading to reduced responses to new antigens and vaccines, increased susceptibility to infections, diminished tumor immunosurveillance, and a chronic, low-grade pro-inflammatory state known as “inflammaging” [28-31].

Several key features characterize immunosenescence and are relevant to LN pathogenesis. Direct pathways linking aging to lymphomagenesis include DNA damage accumulation, telomere attrition, and impaired DNA repair mechanisms [32]. These processes are particularly relevant in the context of viral-associated lymphomas. For instance, Epstein–Barr virus (EBV), which is detectable in approximately 40% of Hodgkin lymphoma cases in Western countries, exploits age-related immune dysfunction [33]. EBV prevalence in HL follows the bimodal age distribution, with higher rates in children and older adults compared to young adults, suggesting age-specific interactions between viral persistence and immune surveillance [34]. Changes occur at the level of hematopoietic stem cells (HSCs), where aging leads to a bias towards myeloid differentiation (myeloid skewing) at the expense of lymphopoiesis, particularly affecting B-cell development [28,35-37]. This shift is partly driven by epigenetic alterations and reduced responsiveness to crucial cytokines like IL-7 [28,38]. Furthermore, the progressive involution of the thymus gland, starting early in life, results in an exponential decline in naive T-cell output [39]. This thymic decline restricts T-cell receptor (TCR) repertoire diversity, thereby impairing the capacity to respond effectively to novel antigens, including tumor antigens [39,40]. Studies show that this decline in TCR diversity occurs later in females, which may partially explain their lower overall cancer incidence [39]. Concurrently, the composition of peripheral lymphocyte populations shifts; older individuals typically show fewer naive T and B cells but a relative increase in memory T cells, often exhibiting features of exhaustion such as the loss of CD28/CD27 co-stimulatory molecules, and certain memory B cell subsets like IgD-CD27-double-negative B cells [41-44]. Functionally, aging lymphocytes display a reduced proliferative capacity, impaired B-cell class-switching and antibody affinity, decreased NK cell cytotoxicity, and altered macrophage polarization that can favor pro-tumorigenic M2 phenotypes [45-47]. Finally, inflammaging (chronic low-grade inflammation driven by senescent cells producing the senescence-associated secretory phenotype (SASP), persistent infections like CMV, and metabolic changes) creates an environment that promotes cancer development [48-50].

Emerging evidence suggests that immunosenescence, particularly the decline driven by thymic involution, may be a more potent driver of age-related cancer incidence, including LN, than the traditionally emphasized accumulation of somatic mutations. The paradigmatic example is Epstein–Barr virus-positive DLBCL (EBV + DLBCL), often termed “DLBCL of the elderly,” where declining immune surveillance against EBV-infected B cells in the context of immunosenescence is thought to be central to its pathogenesis [51,52]. Recent evidence suggests that EBV-positive large B-cell lymphoma in young patients (<45 years) frequently shows PD-L1 overexpression (77%) and preferential nodal involvement with an excellent prognosis, contrasting with the more aggressive clinical features in elderly patients [53]. This age-dependent difference in tumor behavior highlights how immunosenescence influences lymphomagenesis through distinct mechanisms across the lifespan.

## 2. Quantifying Aging: Biological Age and Aging Clocks

While chronological age is associated with immunosenescence and LN risk, significant inter-individual variability exists. Biological age aims to capture this variability, providing a measure of an individual’s physiological state and risk for age-related outcomes [7]. Recent transcriptomic studies have identified universal and specific mechanisms of aging across tissues, providing molecular hallmarks that can quantify both aging and mortality risk [54].

### 2.1. Measures of Biological Age

Biological age can be estimated using various approaches. These include clinical and functional measures like frailty indices, gait speed, grip strength, and cognitive function assessments [7,55]. Molecular biomarkers such as telomere length, inflammatory markers (e.g., CRP, IL-6), and metabolic markers also provide insights [19,56,57]. Furthermore, “omics”-based predictors derived from transcriptomics, proteomics, metabolomics, and notably, epigenomics, offer high-dimensional data for estimating biological age [54,58].

### 2.2. Aging Clocks: Robust Tools for Biological Age Estimation

Aging clocks use molecular data to predict and quantify biological aging. Epigenetic clocks, which track age-related changes in DNA methylation at specific CpG sites, are now the gold standard for measuring biological age [12,14,59]. First-generation clocks, including Horvath’s pan-tissue clock (353 CpGs) and Hannum’s blood-based clock (71 CpGs), primarily predict chronological age [12,14]. Second-generation clocks, such as PhenoAge (513 CpGs linked to phenotypic aging and mortality) and GrimAge (1030 CpGs predicting time-to-death, and incorporating smoking history and plasma protein surrogates), demonstrate stronger associations with healthspan, disease risk, and mortality [15,16]. The discrepancy between the clock’s predicted biological age (e.g., DNAm Age) and an individual’s chronological age is termed “age acceleration,” and is a critical metric linked to adverse health outcomes [17,19]. A 5-year increase in epigenetic age acceleration, for instance, has been associated with approximately a 20% increase in mortality risk [18]. Recent epigenome-wide Mendelian randomization studies have identified causal CpG sites that contribute to aging, distinguishing between damaging and adaptive methylation changes [9].

Beyond epigenetics, other biological clocks have been developed based on diverse inputs (summarized in Table 1). These include the immunity clock, utilizing immune function variables like neutrophil and lymphocyte activity [60]; the inflammatory clock (iAge), based on circulating inflammatory proteins such as CXCL9 [61]; the glycan clock, which uses age-related changes in IgG glycosylation patterns [62]; and hematological clocks derived from parameters in standard blood counts, like the lymphocyte-to-monocyte ratio or white blood cell counts [63,64]. Recent advances have also led to plasma protein-based organ-specific aging models that reveal diseases as accelerated aging of specific organ systems [57]. These various clocks provide quantitative measures that can potentially disentangle the effects of biological aging from chronological age in the context of LN.

### 2.3. Epigenetic Age Acceleration and LN Risk

Emerging research directly links accelerated biological aging, measured by various clocks, to LN risk and behavior.

Several studies have demonstrated that accelerated epigenetic aging is associated with an increased risk of developing LN. Cohort studies utilizing clocks like HorvathAge, HannumAge, PhenoAge, and GrimAge have found significant associations between epigenetic age acceleration and the risk of incident mature B-cell neoplasms, including DLBCL and other NHL subtypes [17,19,20]. Notably, the second-generation clocks, PhenoAge and GrimAge, often exhibit stronger associations with cancer risk compared to first-generation clocks [19]. These findings suggest that accelerated biological aging processes, as captured by DNAm patterns, may precede and actively contribute to the process of lymphomagenesis. Integrative analyses have uncovered specific mechanisms by which aging drives B cell lymphoma, including promoter hypermethylation and age-associated clonal expansions [22,65]. The nature of epigenetic aging involves both co-regulated changes and stochastic components, as revealed by single-cell analyses [66].

Beyond initial risk, biological age metrics are also pertinent to the clinical course and prognosis of LN. In Chronic Lymphocytic Leukemia (CLL), studies indicate that proliferating tumor cell fractions exhibit a higher estimated DNAm Age compared to resting fractions, suggesting an acquisition of longevity-associated methylation changes as the disease progresses [21]. Furthermore, epigenetic classification systems derived from DNAm profiles offer robust prognostic information in CLL, potentially outperforming traditional markers like IGHV mutation status [67,68]. Age also significantly impacts outcomes in marginal zone lymphoma (MZL), with a recent multicenter study demonstrating that patients >70 years had inferior progression-free survival and overall survival despite the development of new therapies [69]. Older MZL patients presented with worse performance status, advanced stage disease, and bone marrow involvement, yet were more likely to receive single-agent rituximab rather than chemoimmunotherapy, highlighting the complex interplay between biological age, treatment tolerance, and outcomes. Researchers have proposed that measuring epigenetic age acceleration during remission could predict relapse risk, though this requires specific investigation in LN subtypes [70].

The relationship between other aging biomarkers and LN is also under investigation. Telomere length, for instance, shows complex associations; some studies report positive correlations between longer leukocyte telomere length and the risk for lymphoid leukemia, multiple myeloma, and NHL, which contrasts with findings for many solid tumors [56,71]. This underscores the necessity for context-specific interpretation of aging biomarkers in different cancer types. While less studied specifically for LN risk compared to epigenetic clocks, measures derived from immunity clocks [60] and inflammatory clocks (iAge) [61] are mechanistically linked to immunosenescence and inflammaging, suggesting their potential relevance for LN development and prognosis. Additionally, the recent identification of lymphoid clonal hematopoiesis of indeterminate potential (L-CHIP) in healthy individuals provides a potential precursor state. Characterized by clonal expansions carrying mutations typically found in LN, L-CHIP is specifically associated with future LN development and may represent an early manifestation of age-related processes culminating in overt malignancy [72]. The relationship between L-CHIP and immunosenescence remains an active area of investigation, with emerging evidence suggesting that age-related immune dysfunction may permit the expansion of these precursor clones [32]. This is supported by findings that age-associated clonal B cells (ACBCs) drive B cell lymphoma development through epigenetic alterations and accelerated biological aging [22].

### 2.4. Clinical Implications and Therapeutic Opportunities

The understanding that biological age, distinct from chronological age, influences LN development and outcomes has significant clinical implications.

Diagnosing LN in older adults is often complicated by the presence of frailty, multiple comorbidities, and atypical clinical presentations [1]. Biological age assessments could significantly complement chronological age and standard prognostic factors. While not a direct aging clock, Comprehensive Geriatric Assessment (CGA) incorporates functional aspects of aging and is crucial for identifying frail patients who may struggle to tolerate standard therapies [29,73]. Sarcopenia, the age- and cancer-related decline in muscle mass and function, is another critical factor. It serves as a powerful predictor of treatment-related toxicity, such as febrile neutropenia, and poorer survival outcomes in LN patients, particularly those with DLBCL [29,74-76]. Objective measurement via CT imaging (e.g., L3 skeletal muscle index, LSMI) facilitates its assessment in clinical practice [74]. Sarcopenia likely reflects aspects of accelerated biological aging and strongly correlates with frailty [77]. Studies have shown that mitochondrial dysfunction, a hallmark of aging, contributes to muscle wasting and may be targeted by interventions such as acarbose that modulate aging pathways [78,79]. Integrating quantitative aging clock data (e.g., DNAm age acceleration) with CGA and sarcopenia measures could potentially lead to more precise risk stratification and better prediction of treatment tolerance in the future. However, practical challenges including the cost and accessibility of DNAm testing remain significant barriers to clinical implementation. While costs have decreased substantially, comprehensive epigenetic profiling still requires specialized equipment and expertise that may not be readily available in all clinical settings [12]. Recent innovations such as TIME-seq have reduced both the time and cost of DNA methylation measurement for epigenetic clock construction [80].

Insights derived from biological age could guide more individualized therapeutic approaches for LN patients. Identifying biologically older or frail individuals, irrespective of their chronological age, could support decisions regarding the use of dose-reduced regimens or alternative therapies, aiming to mitigate toxicity while preserving efficacy [29,73]. As observed in CLL, epigenetic profiles associated with biological aging might predict both treatment response and relapse risk, potentially informing therapy selection [67,70]. Furthermore, for cancer survivors or patients exhibiting signs of accelerated aging, interventions targeting fundamental aging processes (such as senolytics designed to clear senescent cells, or drugs like metformin and resveratrol) are being explored, although rigorous clinical validation in the context of LN is still required [7]. Currently, there are no dedicated clinical trials specifically investigating senolytics or metformin for lymphoid malignancies, representing a significant research gap. These anti-aging agents have the theoretical potential to “reverse aging or at least slow down the accelerated aging process and improve quality of life in cancer survivors during the active treatment and after the treatment ends,” but their specific application to LN requires investigation [7]. Successfully reversing or slowing biological age acceleration could potentially enhance long-term outcomes and improve the quality of life for these patients.

The escalating costs associated with novel LN therapies underscore the need for costeffective management strategies [40,67,81]. Biological age assessment could potentially aid in optimizing resource allocation by helping to identify patients who are most likely to derive significant benefit from intensive or high-cost treatments, versus those for whom supportive or palliative approaches may be more appropriate. The development of standardized platforms for biological age profiling, such as ClockBase, facilitates the clinical implementation of these tools [10].

## 3. Future Directions

Despite significant progress, several areas demand further investigation to fully harness the potential of biological aging insights in LN. A primary need is the refinement and validation of aging clocks specifically tailored to lymphoid tissues or LN subtypes, alongside improving the clinical applicability and accessibility of current clock technologies [8,11]. Recent developments in foundation models for the DNA methylome, such as MethylGPT, offer new opportunities for more accurate and interpretable aging biomarkers [59]. Establishing clear causal links between accelerated biological aging, its drivers like immunosenescence, and LN development is crucial, moving beyond correlative findings through longitudinal studies tracking biological age changes prior to LN diagnosis. Recent advances in causal inference for epigenetic aging, including epigenome-wide Mendelian randomization approaches, provide tools to identify causal methylation sites [9,13].

Further research should delve deeper into the mechanisms by which specific aging pathways, such as the senescence-associated secretory phenotype (SASP), T-cell mitochondrial dysfunction [82,83], or altered HSC function [84], contribute to lymphomagenesis. Studies have shown that protective genetic backgrounds with a reduced burden of damaging variants contribute to exceptional longevity [85], suggesting potential targets for intervention. Prospective clinical trials are essential to evaluate the utility of biological age assessments (integrating clocks, CGA, and sarcopenia) in guiding LN treatment decisions and to test the efficacy of “geroprotective” or anti-aging interventions as adjuncts to standard LN therapy or in high-risk individuals (e.g., those with L-CHIP or significant age acceleration). Integrating multi-omics data (combining epigenetic, transcriptomic, proteomic, and metabolomic clocks with clinical information) will likely provide a more holistic view of biological aging in LN [58,86]. High-dimensional representations of biological aging across functional modules, such as the Ageome framework, enable the comprehensive assessment of aging dynamics within individuals and populations [58]. Specific interventions for thymic rejuvenation warrant investigation, including interleukin-7 (IL-7) recombination therapy, which plays a crucial role in T cell development and homeostasis [87]. Studies have shown that thymic hyperplasia occurs in 28.4% of lymphoma patients after chemotherapy, with a younger age being the strongest predictor of thymic regrowth. Other promising approaches include mTOR inhibition and NAD+ activation to overcome T-cell terminal differentiation, which could enhance adoptive cellular immunotherapies in LN patients. Finally, developing effective strategies to counteract the detrimental aspects of immunosenescence, potentially through restoring TCR diversity, rejuvenating thymic function [88], or modulating inflammaging, represents a significant opportunity for LN prevention and therapy.

## 4. Conclusions

The intersection of lymphoid neoplasms, biological aging, and aging clocks represents a rapidly evolving field with profound clinical implications. Chronological age remains a major risk factor for LN, and is largely mediated through immunosenescence. However, biological age, quantified by increasingly sophisticated aging clocks, provides a more precise measure of an individual’s aging trajectory and vulnerability. Accelerated biological aging is demonstrably linked to increased LN risk and impacts disease progression and potentially treatment outcomes. The complex relationship between aging and immune dysfunction has been further elucidated by studies showing how genetic factors influence COVID-19 risk through aging-related pathways [89], highlighting the broader implications of immunosenescence in disease susceptibility. Integrating assessments of biological age, including aging clocks and clinical measures like frailty and sarcopenia, into oncological practice holds immense promise for personalizing LN management, particularly in the growing elderly population. Future research focused on mechanistic understanding, refining predictive tools, and testing targeted interventions aimed at biological aging pathways will be critical to translate these insights into tangible benefits for patients with lymphoid neoplasms. Open scientific competitions, such as the Biomarkers of Aging Challenge, are driving innovation in aging biomarker development and validation [90].

## Figures and Tables

**Table 1.** Biological clocks developed based on diverse inputs.

Clock Type		Biological Basis	Examples/Key Features	References
Epigenetic Clocks	Age-related changes in DNA methylation (DNAm) at specific CpG sites	1st Gen: HorvathAge (pan-tissue), HannumAge (blood). Predict chronological age. 2nd Gen: PhenoAge (phenotypic aging, mortality), GrimAge (mortality, incorporates biomarkers like smoking). Stronger healthspan association.	High—risk stratification; prognosis in CLL and DLBCL. Requires specialized equipment.	[12,14-16]
Immunity Clock	Functional immune parameters	Neutrophil/lymphocyte chemotaxis, phagocytosis, NK cell activity. Measures immunological aging rate.	Moderate—potential for assessing immunotherapy response. Research stage.	[60]
Inflammatory Clock (iAge)	Levels of circulating inflammatory proteins	Based on markers like CXCL9, eotaxin. Reflects chronic inflammation/inflammaging.	Moderate—may predict therapy-related toxicity. Accessible via blood tests.	[61]
Glycan Clock	Changes in immunoglobulin G (IgG) glycosylation patterns	Reflects alterations in protein glycosylation with age; potentially reversible.	Low—limited LN-specific data. Potential for monitoring treatment effects.	[62]
Hematological Clocks	Parameters from standard blood cell counts	White blood cell counts, lymphocyte-to-monocyte ratio, red cell distribution width. Reflects hematopoietic system aging.	High—readily available from routine labs. Useful for frailty assessment.	[63,64]
